# Supplementary material for: Offspring of first-generation hatchery steelhead trout (Oncorhynchus mykiss) grow faster in the hatchery than offspring of wild fish, but survive worse in the wild: Possible mechanisms for inadvertent domestication and fitness loss in hatchery salmon
Source: PLoS One. 2021 Dec 16;16(12):e0257407. doi: 10.1371/journal.pone.0257407 (PMC8675725; doi:10.1371/journal.pone.0257407)
Supplement: S1 Table — (DOCX) [file pone.0257407.s001.docx]

**S1 Table.**

**A.** Coefficients from **Negative-Binomial** regressions of average size of each family against survival (counts) of each family.

(1) For body size measured under a high fat diet.

| Coefficient | Estimate | Std. Error | z value | Pr(>\|z\|) |
| --- | --- | --- | --- | --- |
| (Intercept) | 4.93571 | 1.61001 | 3.066 | 0.00217 ** |
| Avg.size | -0.01680 | 0.01261 | -1.333 | 0.18265 |
| TypeWxW | 0.25346 | 0.15494 | 1.636 | .10186 |

(2) For body size measured under a low fat diet.

| Coefficient | Estimate | Std. Error | z value | Pr(>\|z\|) |
| --- | --- | --- | --- | --- |
| (Intercept) | 5.46279 | 1.65420 | 3.302 | 0.000959 *** |
| Avg.size | -0.02146 | 0.01325 | -1.619 | 0.105418 |
| TypeWxW | 0.22058 | 0.15952 | 1.383 | 0.166720 |

**B.** Coefficients from **Poisson** regressions of average size of each family against survival (counts) of each family.

(1) For body size measured under a high fat diet.

| Coefficient | Estimate | Std. Error | t value | Pr(>\|t\|) |
| --- | --- | --- | --- | --- |
| (Intercept) | 4.85917 | 1.63864 | 2.965 | 0.00541 ** |
| Avg.size | -0.01623 | 0.01286 | -1.262 | 0.21524 |
| Type WxW | 0.26356 | 0.15851 | 1.663 | 0.10528 |

(2) For body size measured under a low fat diet.

| Coefficient | Estimate | Std. Error | t value | Pr(>\|t\|) |
| --- | --- | --- | --- | --- |
| (Intercept) | 5.33442 | 1.67642 | 3.182 | 0.00306 ** |
| Avg.size | -0.02042 | 0.01346 | -1.517 | 0.13824 |
| Type WxW | 0.22500 | 0.16362 | 1.375 | 0.17781 |
